# Optimal timing for prediction of pathologic complete response to neoadjuvant chemoradiotherapy with diffusion-weighted MRI in patients with esophageal cancer

**DOI:** 10.1007/s00330-019-06513-0

**Published:** 2019-12-10

**Authors:** Alicia S. Borggreve, Sophie E. Heethuis, Mick R. Boekhoff, Lucas Goense, Peter S. N. van Rossum, Lodewijk A. A. Brosens, Astrid L. H. M. W. van Lier, Richard van Hillegersberg, Jan J. W. Lagendijk, Stella Mook, Jelle P. Ruurda, Gert J. Meijer

**Affiliations:** 1 Department of Radiation Oncology, University Medical Center Utrecht, Utrecht University, Heidelberglaan 100, 3584 CX Utrecht, The Netherlands; 2 Department of Surgery, University Medical Center Utrecht, Utrecht University, Heidelberglaan 100, 3584 CX Utrecht, The Netherlands; 3 Department of Pathology, University Medical Center Utrecht, Utrecht University, Heidelberglaan 100, 3584 CX Utrecht, The Netherlands

**Keywords:** Esophageal cancer, Chemoradiotherapy, Neoadjuvant treatment, Diffusion-weighted magnetic resonance imaging, Organ-sparing treatment

## Abstract

**Objective:**

This study was conducted in order to determine the optimal timing of diffusion-weighted magnetic resonance imaging (DW-MRI) for prediction of pathologic complete response (pCR) to neoadjuvant chemoradiotherapy (nCRT) for esophageal cancer.

**Methods:**

Patients with esophageal adenocarcinoma or squamous cell carcinoma who planned to undergo nCRT followed by surgery were enrolled in this prospective study. Patients underwent six DW-MRI scans: one baseline scan before the start of nCRT and weekly scans during 5 weeks of nCRT. Relative changes in mean apparent diffusion coefficient (ADC) values between the baseline scans and the scans during nCRT (ΔADC(%)) were compared between pathologic complete responders (pCR) and non-pCR (tumor regression grades 2–5). The discriminative ability of ΔADC(%) was determined based on the *c*-statistic.

**Results:**

A total of 24 patients with 142 DW-MRI scans were included. pCR was observed in seven patients (29%). ΔADC(%) from baseline to week 2 was significantly higher in patients with pCR versus non-pCR (median [IQR], 36% [30%, 41%] for pCR versus 16% [14%, 29%] for non-pCR, *p* = 0.004). The ΔADC(%) of the second week in combination with histology resulted in the highest *c*-statistic for the prediction of pCR versus non-pCR (0.87). The *c*-statistic of this model increased to 0.97 after additional exclusion of patients with a small tumor volume (< 7 mL, *n* = 3) and tumor histology of the resection specimen other than adenocarcinoma or squamous cell carcinoma (*n* = 1).

**Conclusion:**

The relative change in tumor ADC (ΔADC(%)) during the first 2 weeks of nCRT is the most predictive for pathologic complete response to nCRT in esophageal cancer patients.

**Key Points:**

*• DW-MRI during the second week of neoadjuvant chemoradiotherapy is most predictive for pathologic complete response in esophageal cancer.*

*• A model including ΔADC*_*week 2*_ *was able to discriminate between pathologic complete responders and non-pathologic complete responders in 87%.*

*• Improvements in future MRI studies for esophageal cancer may be obtained by incorporating motion management techniques.*

**Electronic supplementary material:**

The online version of this article (10.1007/s00330-019-06513-0) contains supplementary material, which is available to authorized users.

## Introduction

Neoadjuvant chemoradiotherapy (nCRT) followed by esophagectomy is considered the standard of care for locally advanced, resectable esophageal cancer without distant metastases [1, 2]. Through tumor downsizing and downstaging, nCRT improves locoregional control and overall survival rates compared to surgery alone [2–4]. The degree of tumor regression in response to nCRT is directly related to long-term survival, with pathologic complete response (pCR) resulting in the most favorable long-term prognosis [4, 5]. A pCR, defined as the absence of viable tumor cells at the site of the primary tumor after nCRT, is observed in around 16–29% of the patients after nCRT [2, 6, 7]. For these pathologic complete responders, it is questioned whether they benefit from a subsequent esophagectomy, which is associated with substantial morbidity and impaired quality of life [8–13]. In order to study the safety and feasibility of postponing or even omitting esophagectomy in these patients, accurate prediction of complete responders is essential.

Diffusion-weighted magnetic resonance imaging (DW-MRI) is one of the modalities that is actively studied for its potential in treatment response assessment in multiple cancers, including esophageal cancer [14–20]. DW-MRI is an appealing imaging technique because it is a quantitative method, noninvasive, relatively fast, and without exposure to ionizing radiation [21]. It depends on the microscopic mobility of water and is highly influenced by tissue cellularity and tissue organization [21]. Treatment with chemoradiotherapy can result in the loss of cell membrane integrity and apoptosis, and this process can be detected as an increase in the mean tumor apparent diffusion coefficient (ADC) [22]. However, a subsequent decrease in tumor ADC values may occur by fibrosis, which may complicate interpretation and predictive ability for treatment response [23]. Previous studies have shown promising results for DW-MRI before nCRT, as well as during the first 2–3 weeks of nCRT in the prediction of pathologic response in esophageal cancer patients [14, 15, 24–27]. To investigate and further optimize the predictive ability of DW-MRI during nCRT for response assessment in esophageal cancer, this study aimed at establishing the optimal timing of DW-MRI scanning during nCRT for the prediction of pCR in patients with esophageal squamous cell carcinoma and adenocarcinoma.

## Methods

This single-center, prospective cohort study was approved by the institutional review board of the University Medical Center Utrecht (protocol ID 15-340). All participants provided written informed consent. The primary aim of the study was to research intrafraction tumor motion and regression in order to develop patient-specific adaptive radiotherapy using MRI [28, 29]. The current analysis on the optimal timing for response prediction was a prespecified secondary aim of this prospective study; hence, not all patients who were eligible for inclusion in the prospective study were analyzed in the current analysis.

### Study population

Consecutive patients with histologically confirmed squamous cell carcinoma or adenocarcinoma of the esophagus or gastroesophageal junction who were scheduled to undergo nCRT followed by esophagectomy between December 2015 and April 2018 were eligible for inclusion in the current analysis. Exclusion criteria for enrollment in the study included age < 18 years, previous treatment with thoracic surgery or thoracic radiotherapy, and contraindications for MRI. Exclusion criteria for the current analysis included unexpected distant metastatic disease after nCRT, poor tumor visibility on DW-MRI, or withdrawal from study participation. The primary diagnostic workup consisted of an endoscopy with biopsy for diagnosis, as well as an integrated ^18^F-FDG PET/CT scan for clinical staging.

### Treatment

The neoadjuvant treatment regimen consisted of weekly intravenous administration of carboplatin and paclitaxel for 5 weeks with concurrent radiotherapy (41.4 Gy in 23 fractions of 1.8 Gy, see Supplementary material for details) [2]. Surgical resection consisted of a transthoracic or transhiatal esophagectomy with en-bloc two-field lymphadenectomy and gastric conduit reconstruction with either cervical or intrathoracic anastomosis.

### Histopathological assessment

The surgical resection specimen was assessed by a specialized gastrointestinal pathologist who was blinded for the results of the DW-MRI scans. Patients were staged in accordance with the 7^th^ edition of the Union for International Cancer Control (UICC) [30]. Pathologic tumor regression was reported according to the Mandard system (tumor regression grade [TRG] 1 [pCR, ypT0]: complete response with absence of residual cancer cells; TRG 2: rare residual cancer cells scattered through fibrosis; TRG 3: increase in the number of residual cancer cells, but fibrosis still predominates; TRG 4: residual cancer outgrowing fibrosis; TRG 5: absence of regressive changes) [31]. In the absence of macroscopic tumor, any abnormally appearing tissue was evaluated in order to make an adequate assessment of the presence of residual tumor and the effects of therapy.

### Image acquisition

Patients underwent six sequential MRI scans. One baseline MRI scan was performed at a median of 5 days (interquartile range [IQR] 4–8 days) prior to nCRT in addition to the conventional diagnostic workup. Subsequently, five additional MRI scans were performed weekly during nCRT (see Supplementary Fig. 1 for the study design).

All images were acquired on a 1.5-T Philips Ingenia. Respiratory-triggered transverse anatomical T2-weighted scans (tT2W) and DW-MRI scans with three *b*-values (0, 200, and 800 s/mm^2^) were acquired in coronal planes (see Supplementary methods for details).

### Image analysis

The primary tumor—excluding the lumen—was delineated based on the signal of the baseline DW-MRI scans with a *b*-value of 800 s/mm^2^ using open source software with a semi-automated delineation method (ITK-SNAP, www.itksnap.org) [32, 33], allowing for manual editing by two readers (A.S.B. and S.E.H.) in consensus. The primary contouring was propagated to the DW-MRI scans of the subsequent weeks, followed by manual adjustment by one reader (A.S.B.) based on signal reduction on the b800 DW-MRI scans and tumor regression on the tT2W scans using in-house–developed delineation software [34]. Contouring was performed conservatively to avoid edges of the tumor boundaries, as ADC values at the boundaries might be unreliable due to motion or image distortions [15]. In all cases, the apparent tumor bed was verified based on the tT2W images. Since the in-house–developed delineation software interprets images as 3D volumes, there was no need to generate multiplanar reconstructions. The readers were blinded to patient-related characteristics and clinical outcome in terms of pathologic response.

ADC maps were generated for each slice based on a mono-exponential model fitted on *b*-values of 0, 200, and 800 s/mm^2^, as based on earlier experience [14, 15]. Mean tumor ADC values were extracted from the DW-MRI volume of interests. The relative changes in mean ADC values between the baseline scans and the scans during nCRT were calculated and included in the analyses, as based on previous literature (ΔADC(%) = [mean ADC_week(*n*)_ − mean ADC_baseline_] / mean ADC_baseline_) [14, 15, 24].

### Statistical analysis

Patient and treatment-related characteristics are described as counts with percentages, mean (± standard deviation [SD]), or median (IQR). The median delineated tumor volume on the baseline DW-MRI scan was compared between patients with a pCR and non-pCR using the nonparametric Mann–Whitney *U* test.

In order to determine the optimal timing of DW-MRI scanning for prediction of pCR, the ΔADC(%) was compared between patients with a pCR and non-pCR per week using the Mann–Whitney *U* test. The ability of the ΔADC(%) parameters per week to discriminate between pCR and non-pCR was quantified using ridge regression, including tumor histology as determined on the tumor biopsy—an important known factor to impact pCR. Subsequently, the area under the receiver-operating characteristic (ROC) curve (*c*-statistic) was calculated. Missing ADC values were imputed with using multiple imputation. Subsequently, ΔADC(%) values were calculated and the ridge regression model was fitted on all imputed datasets (see Supplementary material for details).

Furthermore, in order to determine whether the results of future studies could be optimized when applying additional exclusion criteria, a post hoc sensitivity analysis was performed. Patients with small tumor volumes (as the signal blurring caused by respiratory motion is more pronounced in small tumors) as well as patients with a histologic tumor type other than adenocarcinoma or squamous cell carcinoma based on the resection specimen were excluded. The performance measure of interest in this sensitivity analysis was the *c*-statistic.

All statistical analyses were performed using R software for statistical computing version 3.5.1 (‘mice’ [35], ‘glmnet’ [36], and ‘Hmisc’ [37] packages, www.R-project.org). The significance level was set at *p* < 0.05. No corrections for multiple testing were performed, as the universal null hypothesis was not of interest [38]. Furthermore, as this study was of descriptive nature, no formal power calculation was performed.

## Results

### Patients

A total of 32 patients with newly diagnosed esophageal cancer were enrolled in the prospective study. Of these patients, 8 were excluded for the current analysis based on unexpected distant metastatic disease after nCRT (*n* = 2), tumor histology other than squamous cell carcinoma or adenocarcinoma as based on the primary tumor biopsy (*n* = 2), poor tumor visibility on DW-MRI (*n* = 3), or withdrawal from study participation (*n* = 1) (Supplementary Fig. 2). The final study population comprised 24 patients with a mean age of 65 years (± 8 years) and all but 2 were male (92%). The majority of the patients had an adenocarcinoma (67%). Median time between nCRT and esophagectomy was 10 weeks (IQR 7–14 weeks). A pCR (TRG 1) after nCRT was observed in 7 patients (29%). Table 1 gives an overview of the clinical characteristics of the study population.

**Table 1:** Clinical characteristics of the study population

Characteristic	Full cohort (*n* = 24)		pCR (*n* = 7)		Non-pCR (*n* = 17)	
	*n*	(%)	*n*	(%)	*n*	(%)
Age at diagnosis, in years (mean ± SD)	65 ± 8		68 ± 7		64 ± 8	
Sex						
Male	22	92%	7	100%	15	88%
Female	2	8%	0	0%	2	12%
Tumor location						
Middle esophagus	1	4%	0	0%	1	6%
Distal esophagus	22	92%	7	100%	15	88%
Gastroesophageal junction	1	4%	0	0%	1	6%
Clinical T stage*						
cT2	1	4%	0	0%	1	6%
cT3	23	96%	7	100%	16	94%
Clinical N stage*						
cN0	8	33%	3	43%	5	29%
cN1	13	54%	2	29%	11	65%
cN2	2	8%	2	29%	0	0%
cN3	1	4%	0	0%	1	6%
Histologic tumor type (biopsy)						
Adenocarcinoma	16	67%	3	43%	13	76%
Squamous cell carcinoma	8	33%	4	57%	4	24%
Tumor regression grade (TRG)						
TRG 1 (pCR)	7	29%	7	100%	NA	
TRG 2	6	25%	NA		6	35%
TRG 3	7	29%	NA		7	41%
TRG 4	3	13%	NA		3	18%
TRG 5	1	4%	NA		1	6%
Pathological T stage*						
ypT0	7	29%	7	100%	NA	
ypT1	3	13%	NA		3	18%
ypT2	6	25%	NA		6	35%
ypT3	8	33%	NA		8	47%
Pathological N stage*						
ypN0	15	63%	6	86%	9	53%
ypN1	4	17%	1	14%	3	18%
ypN2	4	17%	0	0%	4	24%
ypN3	1	4%	0	0%	1	6%
Surgical approach						
Thoracolaparoscopic	18	75%	5	71%	13	76%
Laparoscopic transhiatal	5	21%	1	14%	4	24%
Open transthoracic	1	4%	1	14%	0	0%
Lymph node yield (median, IQR)	30	(19–36)	29	(23–40)	30	(19–35)
Positive lymph nodes harvested (median, IQR)	0	(0–1)	0	(0–0)	1	(0–3)

*IQR* interquartile range; *NA* not applicable; *pCR* pathologic complete response; *SD* standard deviation; *TRG* tumor regression grade

*Clinical and histopathologic T- and N-stage are based on UICC TNM 7th edition

All patients received 5 cycles of chemotherapy and the full course of radiation therapy. In one patient, it was decided during nCRT to extend the regimen with 1 week, to a total dose of 50.4 Gy and 6 cycles of chemotherapy. Pretreatment DW-MRI scans were available in all patients. Two DW-MRI scans during nCRT were missing due to patient’s refusal (*n* = 1) or image acquisition problems (*n* = 1), resulting in a total of 142 MRI scans.

The median delineated tumor volume on the baseline DW-MRI scan was 15 mL (IQR 11–23 mL) and did not significantly differ between pCR and non-pCR patients (median [IQR]: 11 mL [7–22 mL] versus 16 mL [11–23 mL], respectively, *p* = 0.318).

### ADC changes during nCRT

**Fig. 1:**
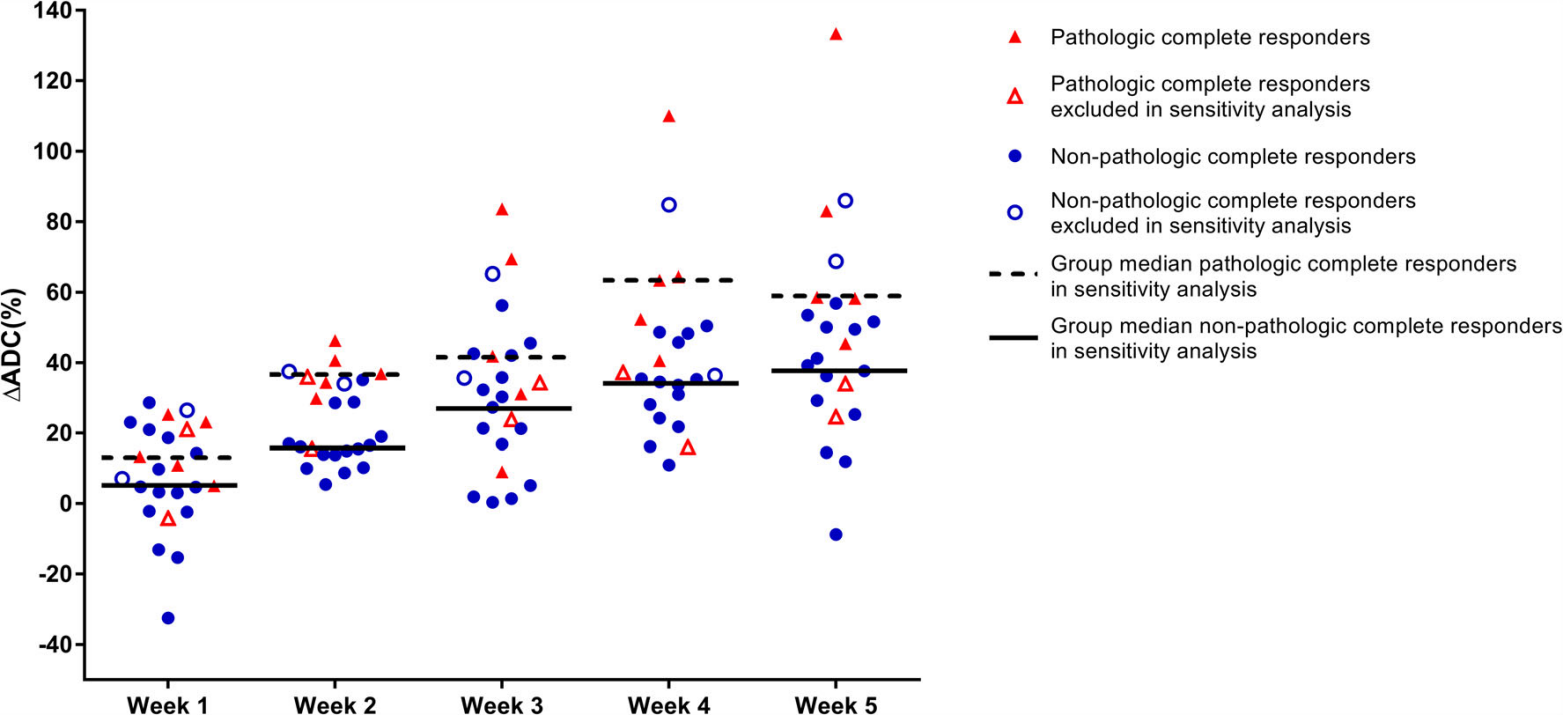
Relative changes in ADC values between baseline scans and scans during neoadjuvant chemoradiotherapy between pathologic complete responders (pCR, red triangles) and poor responders (non-pCR, blue circles). Patients who were excluded in the post hoc sensitivity analysis are marked with an empty symbol.

**Fig. 2:**
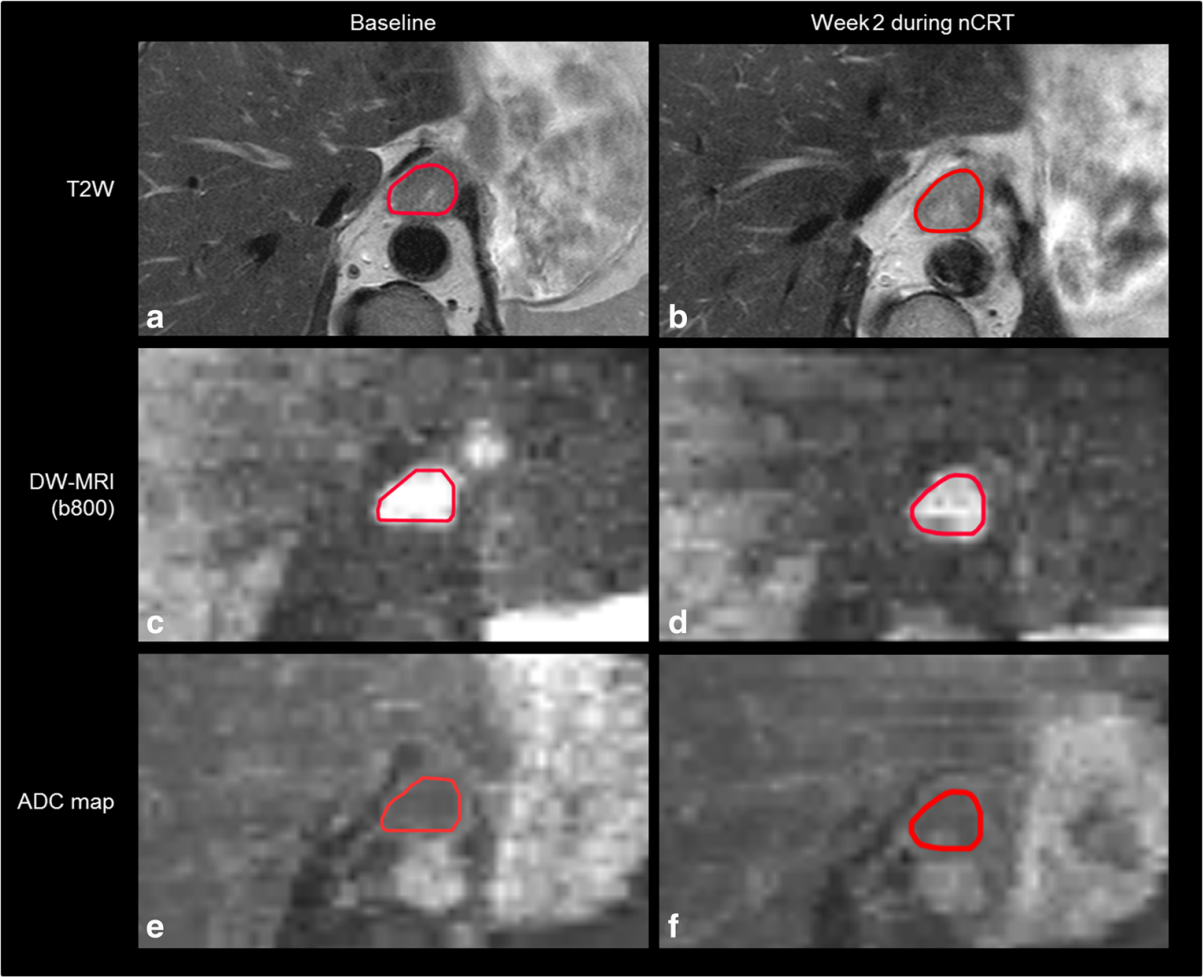
Patient with a cT3N2M0 distal esophageal squamous cell carcinoma with a pathologic complete response (pCR, TRG 1) to neoadjuvant chemoradiotherapy and a ΔADC_week 2_ of 29%. T2 weighted images (**a** and **b**), diffusion-weighted images (*b*-value = 800 s/mm^2^) (**c** and **d**), and ADC maps (**e** and **f**) on a 1.5-T MR scanner before (**a**, **c**, **e**) and in the second week of neoadjuvant chemoradiotherapy (**b**, **d**, **f**)

The relative increase in tumor ADC from baseline DW-MRI scans to scans acquired in the second week of nCRT (ΔADC_week 2_) was significantly associated with pCR (median [IQR]: 36% [30–41%] for pCR versus 16% [14–29%] for non-pCR, *p* = 0.004). In contrast, relative changes in ADC from baseline to DW-MRI scans acquired in the other weeks of nCRT were not significantly different between pCR and non-pCR groups (Table 2, Fig. 1). Figure 2 presents baseline MRI scans and MRI scans in the second week of nCRT of a patient with pCR.

**Table 2:** Relative changes in ADC per week during neoadjuvant chemoradiotherapy between esophageal cancer patients with a pathologic complete response and non-pathologic complete response

		Median ΔADC (%) (IQR)		*p* value*
		pCR	Non-pCR	
Full cohort (*n* = 24)	Week 1	13 (5, 23)	5 (− 2, 19)	0.260
	Week 2	36 (30, 41)	16 (14, 29)	0.004
	Week 3	34 (24, 69)	30 (17, 42)	0.318
	Week 4	52 (37, 64)	35 (26, 47)	0.065
	Week 5	58 (34, 83)	40 (27, 53)	0.198
Sensitivity analyses (*n* = 20)^✝^	Week 1	13 (11, 23)	5 (− 2, 19)	0.098
	Week 2	37 (24, 41)	16 (10, 19)	0.001
	Week 3	42 (31, 69)	27 (5, 42)	0.168
	Week 4	63 (52, 64)	34 (24, 46)	0.002
	Week 5	59 (58, 83)	38 (25, 50)	0.003

*ADC* apparent diffusion coefficient; *IQR* interquartile range; *pCR* pathologic complete response

*p value based on Mann–Whitney *U* test

^✝^After post-hoc exclusion of additional four patients based on baseline tumor volume delineated on DW-MRI < 7 mL (*n* = 3) and tumor histology other than adenocarcinoma or squamous cell carcinoma as based on the resection specimen (*n* = 1)

**Fig. 3:**
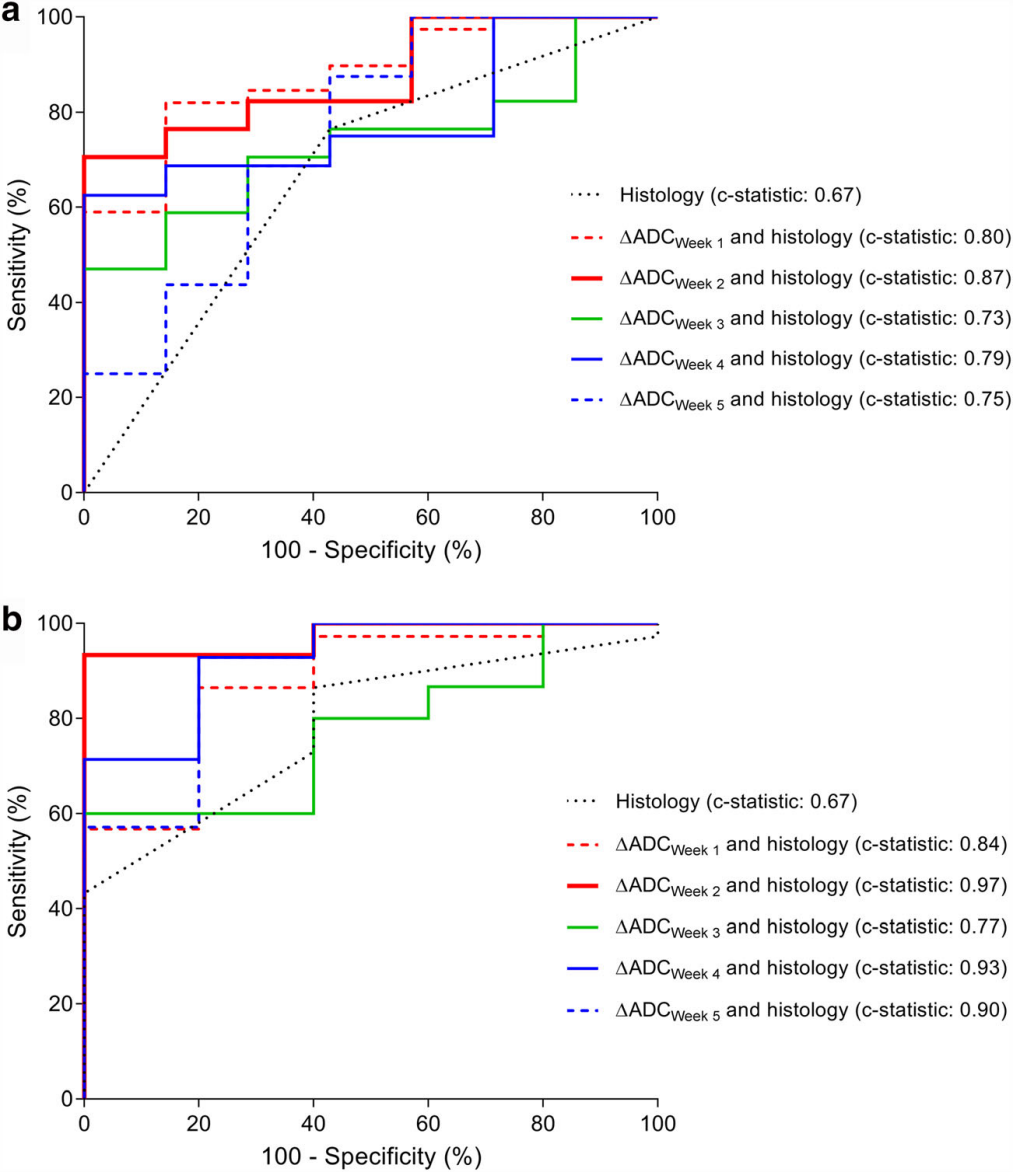
Receiver-operating characteristic curve analysis for the regression models with relative changes in ADC per week, as well as histopathological tumor type, for discriminating between pCR and non-pCR patients in the full cohort (**a**) as well as in the sensitivity analysis (**b**)

**Fig. 4:**
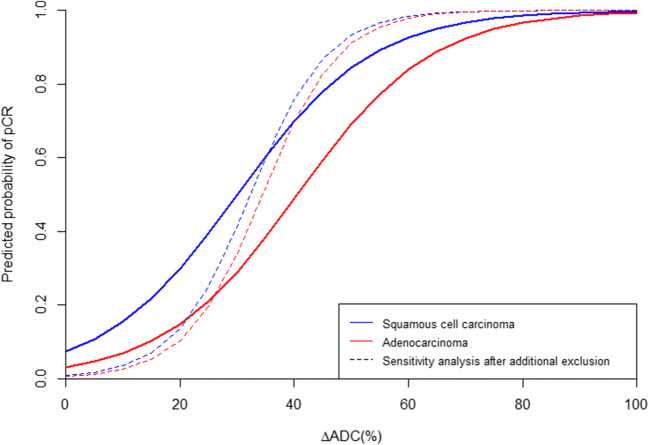
Predictive probability plot for pathologic complete response (pCR) based on the fitted regression model including relative changes in ADC (ΔADC(%)) from baseline to week 2 for squamous cell carcinomas (blue) and adenocarcinomas (red). The dashed lines represent the probability plot for the sensitivity analysis after additional exclusion of 4 patients

ROC curve analyses after ridge-penalized regression analyses taking histology into account demonstrated the highest *c*-statistic of 0.87 for the relative ADC increase from baseline to week 2 of nCRT (ΔADC_week 2_) combined with histology. Poorer discriminative ability was observed for histology alone (*c*-statistic 0.67) or histology in combination with the ΔADC(%) of the other weeks (*c*-statistics 0.73–0.80, Table 3, Fig. 3). A predictive probability plot for pCR based on ΔADC(%) from baseline to week 2 for squamous cell carcinomas and adenocarcinomas is presented in Fig. 4, demonstrating an increase in the probability for pCR when ΔADC(%) increases.

**Table 3:** Ridge regression analyses demonstrating the discriminatory value of DW-MRI parameters per week with pathologic complete response (TRG 1) as outcome variable

Intercept and predictors	Full cohort (*n* = 24)			Sensitivity analyses (*n* = 20)*		
	*β*	OR	*c*-statistic	*β*	OR	*c*-statistic
Histology						
Intercept	− 1.00		0.67	− 1.28		0.67
Squamous cell carcinoma^✝^	0.33	1.39		0.49	1.63	
Week 1						
Intercept	− 1.74		0.80	− 2.30		0.84
ΔADC_week 1_ (%)	0.04	1.04		0.06	1.06	
Squamous cell carcinoma^✝^	1.29	3.63		1.44	4.24	
Week 2						
Intercept	− 3.45		0.87	− 5.17		0.97
ΔADC_week 2_ (%)	0.09	1.09		0.15	1.16	
Squamous cell carcinoma^✝^	0.89	2.44		0.32	1.37	
Week 3						
Intercept	− 1.09		0.73	− 1.76		0.77
ΔADC_week 3_ (%)	0.00	1.00		0.02	1.02	
Squamous cell carcinoma^✝^	0.20	1.23		0.44	1.56	
Week 4						
Intercept	− 1.61		0.79	− 4.76		0.93
ΔADC_week 4_ (%)	0.01	1.01		0.08	1.08	
Squamous cell carcinoma^✝^	0.50	1.65		0.16	1.18	
Week 5						
Intercept	− 1.57		0.75	− 4.03		0.90
ΔADC_week 5_ (%)	0.01	1.01		0.06	1.06	
Squamous cell carcinoma^✝^	0.69	1.99		0.64	1.90	

Note: Due to rounding, the reported odds ratios might not precisely correspond with the reported beta regression coefficients

*ADC* apparent diffusion coefficient, *c-statistic* concordance statistic, *OR* odds ratio, *pCR* pathologic complete response

*After post hoc exclusion of additional four patients based on baseline tumor volume delineated on DW-MRI < 7 mL (*n* = 3) and tumor histology other than adenocarcinoma or squamous cell carcinoma as based on the resection specimen (*n* = 1)

^✝^Adenocarcinoma was used as reference category

### Sensitivity analyses

For the post hoc sensitivity analysis, an additional 4 patients were excluded based on a delineated tumor volume on the baseline DW-MRI scan < 7 mL (*n* = 3) and tumor histology other than adenocarcinoma or squamous cell carcinoma as based on the resection specimen (*n* = 1). In this study population of 20 patients, 5 patients had a pCR of which 2 had an adenocarcinoma and 3 had a squamous cell carcinoma. Exclusion of these additional 4 patients resulted in significant differences between patients with pCR and non-pCR in ΔADC(%) from baseline to weeks 2, 4, and 5 (Table 2). Furthermore, the *c*-statistics improved for the regression models with ΔADC(%) of weeks 1, 2, 3, 4, and 5 to 0.84, 0.97, 0.77, 0.93, and 0.90, respectively (Table 3).

Multiple imputation of the missing ADC value of week 4 and week 5 did not substantially impact the results of the regression analysis in terms of the observed *c*-statistics in the entire cohort (0.80 and 0.72 in the imputed datasets compared to 0.79 and 0.75 in the complete case datasets, respectively) or in the sensitivity analysis (0.93 and 0.87 in the imputed dataset compared to 0.93 and 0.90 in the complete case dataset, respectively) (Supplementary Table 2).

## Discussion

This prospective study was designed to assess the optimal timing of DW-MRI scanning during nCRT for prediction of pCR in esophageal cancer patients. The relative change in ADC (ΔADC) during the first 2 weeks of nCRT demonstrated the highest predictive ability for pCR at the time of surgery. This is of important clinical value as early response evaluation could enable individualized treatment regimens. Accurate assessment of response to nCRT is not only important for safe implementation of an organ-sparing approach in patients with pCR, but also to adapt treatment strategies in expected poor responders. Improving the accuracy of response evaluation after nCRT may provide improved outcomes for both patient groups.

In order to improve patient-friendliness of disease monitoring and (re)staging procedures, it is important to minimize the burden of these procedures. As demonstrated before, DW-MRI is generally well-tolerated by patients, although shorter acquisition times as well as altered body positioning could further improve patient experience [39]. Our results could further aid the minimization of the burden of repeated scanning for patients, as well as optimal usage of the available (financial) resources, while assuring the best predictive ability of DW-MRI.

Previous studies focusing on DW-MRI scanning in response prediction for esophageal cancer have mostly demonstrated promising findings regarding the predictive value of ΔADC(%) for response prediction to nCRT [14, 15, 18, 40, 41]. The majority of these studies reported significant differences between responders and nonresponders in ΔADC(%) from baseline to during nCRT [14, 15, 24, 41], whereas others report significant differences in ΔADC(%) from baseline to follow-up after nCRT [18, 41]. Nevertheless, not all studies found the same predictive effect size, nor did they all report a significant relation between ΔADC(%) and response [42, 43]. Several factors may account for these differences. First, ADCs were calculated based on different *b*-values varying from 0 and 1000 [41], 0 and 600 [24] to 0, 200, and 800 [14, 15]. The choice of *b*-values and the number of signal averages (NSAs) per *b*-value, as well as the calculation of ADC values by various modeling strategies, are known to impact tumor ADC estimates [21]. Second, delineation methods for determining the volume of interest differ. Some studies consider the entire tumor volume as volume of interest [14, 15, 18], whereas others only delineated the tumor on the most representative tumor slice [41]. Lastly, the nCRT regimen of choice varied between the studies.

Similar to our study, Wang et al [24] performed weekly DW-MRI scanning during CRT in esophageal cancer patients to determine the optimal timing of response evaluation with DW-MRI. They also demonstrated DW-MRI scanning in the second or third week of CRT to be optimal for response assessment. As their study included only esophageal squamous cell carcinomas, used imaging response criteria as a reference standard (i.e., RECIST criteria [44] that assessed 52% of the patients to be a complete responder) instead of histopathology, used a different nCRT regimen (chemotherapy consisting of cisplatin with either 5-fluorouracil or paclitaxel and radiotherapy consisting of 60 Gy in 30 fractions), and did not use ΔADC(%) but only single time point ADC values as measured, similar findings might not have been expected. Additionally, earlier studies by our group as well as the University of Texas MD Anderson Cancer Center that performed DW-MRI in the second or third week found these ΔADC(%) values to be highly predictive of response to nCRT [14, 15]. Together, this supports the robustness of the findings from the current study.

The interobserver reproducibility of tumor delineation on DW-MRI and ADC measurements in esophageal cancer was shown to be very good by two previous studies, especially for the semi-automated volumetric measurement method (intraclass correlation coefficient 0.96, 95% CI 0.91–0.98, *p* < 0.001), which was also applied in the current study [15, 43]. One of these studies compared manual delineation of a region of interest on the most representative tumor slice to semi-automatic delineation of the whole tumor volume and found negligible differences in mean ADC measurements (between − 0.25 and 0.31%) [15]. A voxel-based analytical method, where changes in individual voxels can be monitored, may even provide more reliable results [43]. However, such an approach is challenging, since tumor regression is observed during nCRT and the esophagus is a moving organ [28], making spatial registration of DW-MRI obtained before and after start of nCRT complicated.

To improve external validity of our results, no cutoff values for ADC or ΔADC measurements for classification of complete responders versus nonresponders are reported in our study. Cutoff values are likely to be highly influenced by delineation techniques and determination of a 2D or 3D region of interest, as well as the *b*-values on which ADCs are calculated. To demonstrate this, we highlighted previously reported significant cutoff values for ΔADC(%), ADC_mean_, and ADC_median_ for discrimination of responders versus nonresponders in the data of the current study in Supplementary Fig. 3.

A recent meta-analysis demonstrated a pooled AUC of 0.91 (95% CI 0.89–0.94) of ΔADC(%) values for treatment response prediction in esophageal cancer based on four studies [40]. We were not able to reproduce these results in our full cohort, but this cohort included three patients with small tumor volumes (< 7 mL) and one patient who had a mixed adenoneuroendocrine carcinoma (MANEC) upon histopathological evaluation of the resection specimen. As MANECs are known to respond well to nCRT during treatment (which is reflected by an increase in ADC in the first weeks) but progress rapidly after completion, the inclusion of this patient clearly influenced the obtained results in this relatively small cohort [45, 46]. Exclusion of the aforementioned patients dramatically improved the performance of ΔADC(%) for pCR prediction, resulting in a *c*-statistic of 0.97 for week 2. This also demonstrated that the predictive value of relative changes in ADC seems decreased for small tumors, which might be explained by the respiratory movement of the tumor during the DW-MRI scan. Typically, respiratory motion amplitudes of 1–2 cm are observed during scanning [28, 47], which might negatively impact the quantitative ADC assessment especially in small tumors. Improvement in pCR prediction in future studies may be obtained by incorporating motion management techniques.

Previous work has also demonstrated additional value of DCE-MRI, as well as PET–CT scanning in the prediction of treatment response in esophageal cancer patients [15, 48–52]. Furthermore, emerging biomarkers such as circulating tumor DNA might further improve the predictive performance and might contribute to the safe investigation of an organ-sparing approach for predicted pCR to nCRT.

Significant strengths of the current study include the consistent use of one nCRT regimen for all patients, the presence of a histopathologic reference standard in all patients, the inclusion of both squamous cell carcinomas and adenocarcinomas, and the consistent delineation by semi-automatic contouring. However, the specific hardware characteristics of the MR scanner and scan sequences, as well as the applied delineation technique and calculation of the ADC map by a mono-exponential model based on three *b*-values, may limit the generalizability of the results. In addition, whole-tumor summary statistics (such as the mean ADC) are easily applicable, but fail to fully address the important issue of tumor heterogeneity [21]. Furthermore, the relatively small study population might have led to false-negative results (type II error) for differences in ΔADC(%) between responders and nonresponders from baseline to the other weeks than week 2. Moreover, patients were included during a rather long study period of 2.5 years, since many eligible patients refused participation in this demanding and time-consuming study with weekly MRI scanning. However, the patients generally tolerated the MRI scans well (only 3 patients canceled 1 MRI scan during their treatment in the entire cohort of 32 patients) and no adverse events occurred. Lastly, DW-MRI scanning is currently not routinely used in the staging of patients with esophageal cancer, which challenges the direct implementation of the results in clinical practice.

Future comparative studies should focus on further improving response evaluation after nCRT. In this regard, the recently started Dutch multicenter PRIDE study will further investigate the findings of the DW-MRI pilot studies in a larger patient cohort and aims at developing a multimodal clinically applicable prediction model [53].

In conclusion, treatment-induced change in tumor ADC as measured on DW-MRI during the second week is most predictive for pCR to nCRT in esophageal squamous cell carcinoma and adenocarcinoma.

## Electronic Supplementary materials

ESM 1 Supplementary methods (radiotherapy, image acquisition details, statistical analyses). Supplementary Table 1. Image acquisition details. Supplementary Table 2. Ridge regression analyses demonstrating the discriminatory value of DW-MRI parameters per week with pathologic complete response (TRG 1) as outcome variable after multiple imputation (20 datasets) of the missing ADC values in week 4 and 5. Supplementary Figure 1. Study design. Supplementary Figure 2. Flowchart. Supplementary Figure 3. Graphic illustration of ΔADC(%) and ADC cut-off points derived from the available literature (represented by the dashed and solid horizontal lines) on DW-MRI in pathologic complete response assessment for esophageal cancer and applied to the current data. (DOCX 277 KB)

## Abbreviations

ADC: Apparent diffusion coefficient
CTV: Clinical target volume
DW-MRI: Diffusion-weighted magnetic resonance imaging
GTV: Gross target volume
IQR: Interquartile range
MANEC: Mixed adenoneuroendocrine carcinoma
nCRT: Neoadjuvant chemoradiotherapy
NSA: Number of signal averages
pCR: Pathologic complete response
PTV: Planning target volume
ROC: Receiver-operating characteristic
SD: Standard deviation
TRG: Tumor regression grade
tT2W: Transverse anatomical T2-weighted
UICC: Union for International Cancer Control
