# Supplementary material for: Optimal timing for prediction of pathologic complete response to neoadjuvant chemoradiotherapy with diffusion-weighted MRI in patients with esophageal cancer
Source: Eur Radiol. 2019 Dec 10;30(4):1896–907. doi: 10.1007/s00330-019-06513-0 (PMC7062655; doi:10.1007/s00330-019-06513-0)

**Supplementary material**

**Supplement to:** Borggreve AS, Heethuis SE, Boekhoff MR, et al. Optimal timing for prediction of pathologic complete response to neoadjuvant chemoradiotherapy with diffusion-weighted MRI in patients with esophageal cancer.

**Contents**

**Methods**

*Radiotherapy*

*Image acquisition details*

*Statistical analyses*

**References**

**Supplementary Table 1.** Image acquisition details.

**Supplementary Table 2.** Ridge regression analyses demonstrating the discriminatory value of DW-MRI parameters per week with pathologic complete response (TRG 1) as outcome variable after multiple imputation (20 datasets) of the missing ADC values in week 4 and 5.

**Supplementary Figure 1.** Study design.

**Supplementary Figure 2.** Flowchart.

**Supplementary Figure 3.** Graphic illustration of ΔADC(%) and ADC cut-off points derived from the available literature (represented by the dashed and solid horizontal lines) on DW-MRI in pathologic complete response assessment for esophageal cancer and applied to the current data.

**Methods**

*Radiotherapy*

All patients were treated with volumetric modulated arc radiotherapy. The gross target volume (GTV) was contoured based on information from the endoscopy and ^18^F-FDG PET/CT scan. The extension of the GTV to the clinical target volume (CTV) was typically 3 cm cranially and caudally, and 0.5 cm circumferential without violation of anatomic boundaries. The planning target volume (PTV) expansion was 1 cm isotropically.

*Image acquisition details*

All images were acquired on a 1.5T Philips Ingenia (Best, the Netherlands), using anterior/posterior (28-channel) receive coils. Patients were positioned in supine position with both arms next to the body. Respiratory-triggered transverse anatomical T2-weighted scans (tT2W) were acquired with a multi-slice turbo spin echo sequence (TR/TE = 1604/100ms, resolution = 0.67x0.67x6.48mm^3^). DW-MRI scans with 3 b-values (0, 200 and 800 s/mm^2^) were acquired in coronal planes (number of squared averages [NSA] 4, 3 and 4 for b=0, 200 and 800 s/mm^2^, respectively), using SPIR fat suppression and EPI read-out (resolution = 3.25x3.25x4mm^3^, bandwidth per pixel = 22.9Hz) (see Supplementary Table 1). Images were obtained in coronal planes to mitigate the effects of respiratory motion on image quality, as it was demonstrated before that movement of the esophagus primarily occurs in the cranio-caudal direction.[1] As such, no out-of-plane motion will occur between the diffusion preparation and the image read-out.

*Statistical analyses*

Ridge regression is a penalized regression method, enabling reduction of model overfitting in a situation with few events per variable.[2] The applied overall penalty (λ_min_) represents the minimum mean cross-validated error, which was obtained using 10-fold cross validation.

Missing ADC values were imputed with using multiple imputation (multivariate imputation by chained equations, 20 imputed datasets with a maximum number of 20 iterations for each imputation).[3, 4] All variables in Table 1 as well as the ADC statistics (mean, SD) of all weeks were used as predictor variables for the imputation. Subsequently, ΔADC(%) values were calculated and the ridge regression model was fitted on all imputed datasets. Model coefficients and the c-statistic were pooled using Rubin’s rules.[5]

**References**

1. Lever FM, Lips IM, Crijns SPM, et al (2014) Quantification of Esophageal Tumor Motion on Cine-Magnetic Resonance Imaging. Int J Radiat Oncol Biol Phys 88:419–424. https://doi.org/10.1016/j.ijrobp.2013.10.036

2. Pavlou M, Ambler G, Seaman SR, et al (2015) How to develop a more accurate risk prediction model when there are few events. BMJ 351:h3868. https://doi.org/10.1136/BMJ.H3868

3. Sterne JAC, White IR, Carlin JB, et al (2009) Multiple imputation for missing data in epidemiological and clinical research: potential and pitfalls. BMJ 338:b2393. https://doi.org/doi: 10.1136/bmj.b2393.

4. White IR, Royston P, Wood AM (2011) Multiple imputation using chained equations: Issues and guidance for practice. Stat Med 30:377–399. https://doi.org/10.1002/sim.4067

5. Rubin D (1987) Multiple Imputation for Nonresponse in Surveys. John Wiley & Sons Inc, Hoboken, New Jersey

| **Supplementary Table 1.** Image acquisition details. | | |
| --- | --- | --- |
| **General scanner characteristics** | | |
| Magnetic field strength | | 1.5 T |
| Vendor | | Philips |
| Type | | Ingenia |
|  |  |  |
| **Protocol** | | |
| tT2W | Scan plane | transverse |
|  | Scan mode | Multi-Slice |
|  | Repetition time (ms) | 1604 |
|  | Echo time (ms) | 100 |
|  | Voxel size (mm) | 0.67 |
|  | Slice thickness (mm) | 6.48 |
|  | Flip angle | 90 |
|  | Navigator | Yes |
|  |  |  |
| DWI | Scan plane | Coronal |
|  | Scan mode | Multi-Slice |
|  | Repetition time (ms) | 4299 |
|  | Echo time (ms) | 70 |
|  | Voxel size (mm) | 3.25 |
|  | Slice thickness (mm) | 4.0 |
|  | Flip angle | 90 |
|  | b-values | 0 / 200 / 800 |
|  | Number of squared averages | 4 / 3 / 4 |
|  | Fat suppresion | SPIR |
|  | Navigator | No |

| **Supplementary Table 2.** Ridge regression analyses demonstrating the discriminatory value of DW-MRI parameters per week with pathologic complete response (TRG 1) as outcome variable after multiple imputation (20 datasets) of the missing ADC values in week 4 and 5. | | | | | | | | | |
| --- | --- | --- | --- | --- | --- | --- | --- | --- | --- |
| **Full cohort (n=24)** | | | |  | | **Sensitivity analyses (n=20)*** | | | |
| **Intercept and predictors** | **β** | **OR** | **c-statistic** |  | | **Intercept and predictors** | **β** | **OR** | **c-statistic** |
| **Week 4** | | | | | | | | | |
| Intercept | -1.88 |  | 0.80 | |  | Intercept | -4.64 |  | 0.93 |
| ΔADC_week4_ (%) | 0.02 | 1.02 |  | |  | ΔADC_week4_ (%) | 0.08 | 1.08 |  |
| Squamous cell carcinoma^✝^ | 0.65 | 1.92 |  | |  | Squamous cell carcinoma^✝^ | 0.21 | 1.23 |  |
| **Week 5** | | | | | | | | | |
| Intercept | -1.69 |  | 0.72 |  | | Intercept | -3.24 |  | 0.87 |
| ΔADC_week5_ (%) | 0.01 | 1.01 |  |  | | ΔADC_week5_ (%) | 0.04 | 1.04 |  |
| Squamous cell carcinoma^✝^ | 0.66 | 1.94 |  |  | | Squamous cell carcinoma^✝^ | 0.36 | 1.43 |  |
| *ADC* apparent diffusion coefficient; *c-statistic* concordance statistic; *OR* odds ratio; *pCR* pathologic complete response  Note. Due to rounding, the reported odds ratios might not precisely correspond with the reported beta regression coefficients.  * After post-hoc exclusion of additional 4 patients based on baseline tumor volume delineated on DW-MRI <7ml (n=3) and tumor histology other than adenocarcinoma or squamous cell carcinoma as based on the resection specimen (n=1).  ^✝^Adenocarcinoma was used as reference category. | | | | | | | | | |

**Supplementary Figure 1.** Study design.

**
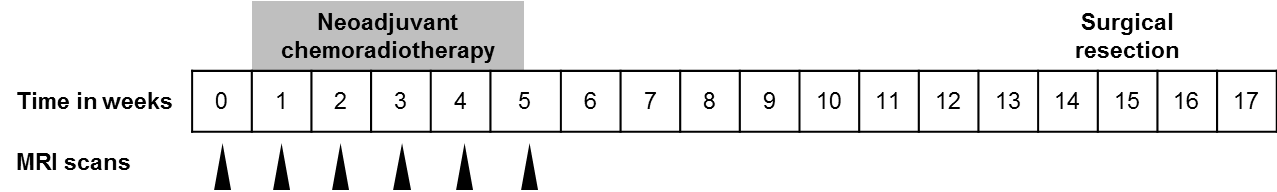
**

**Supplementary Figure 2.** Flowchart.


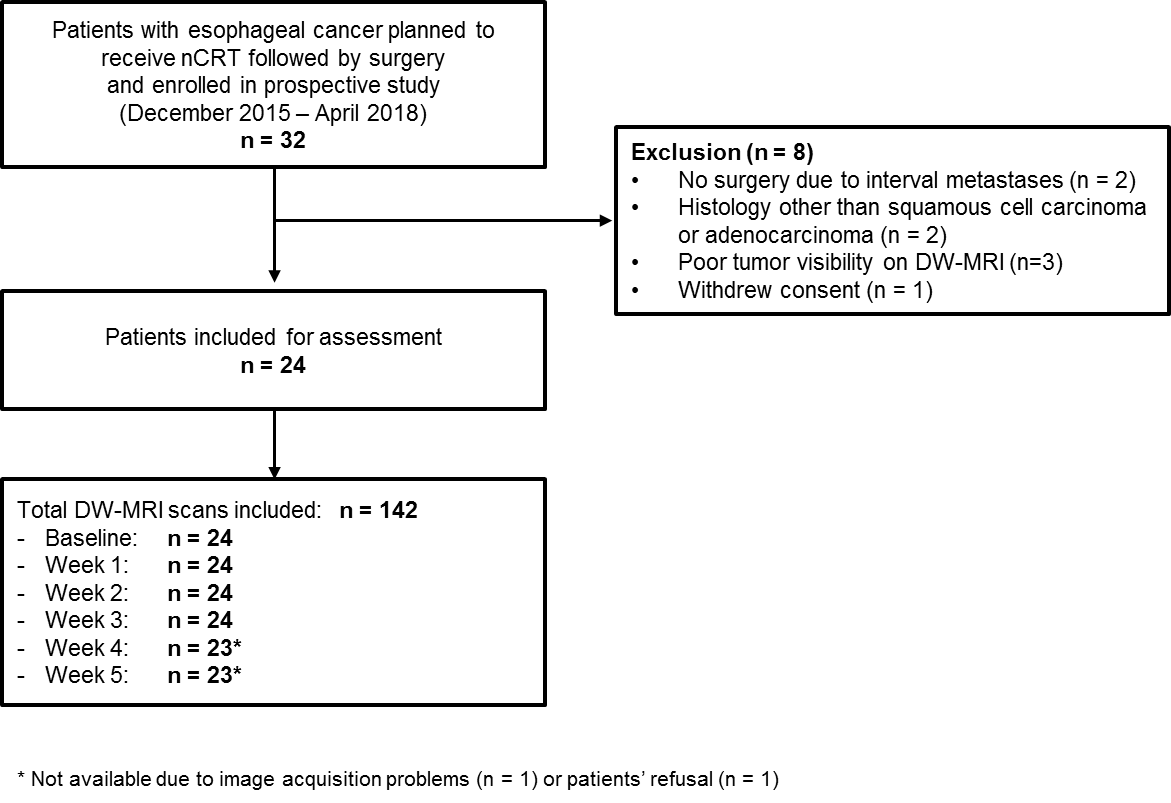


**Supplementary Figure 3.** Graphic illustration of ΔADC(%) and ADC cut-off points derived from the available literature (represented by the dashed and solid horizontal lines) on DW-MRI in pathologic complete response assessment for esophageal cancer and applied to the current data.


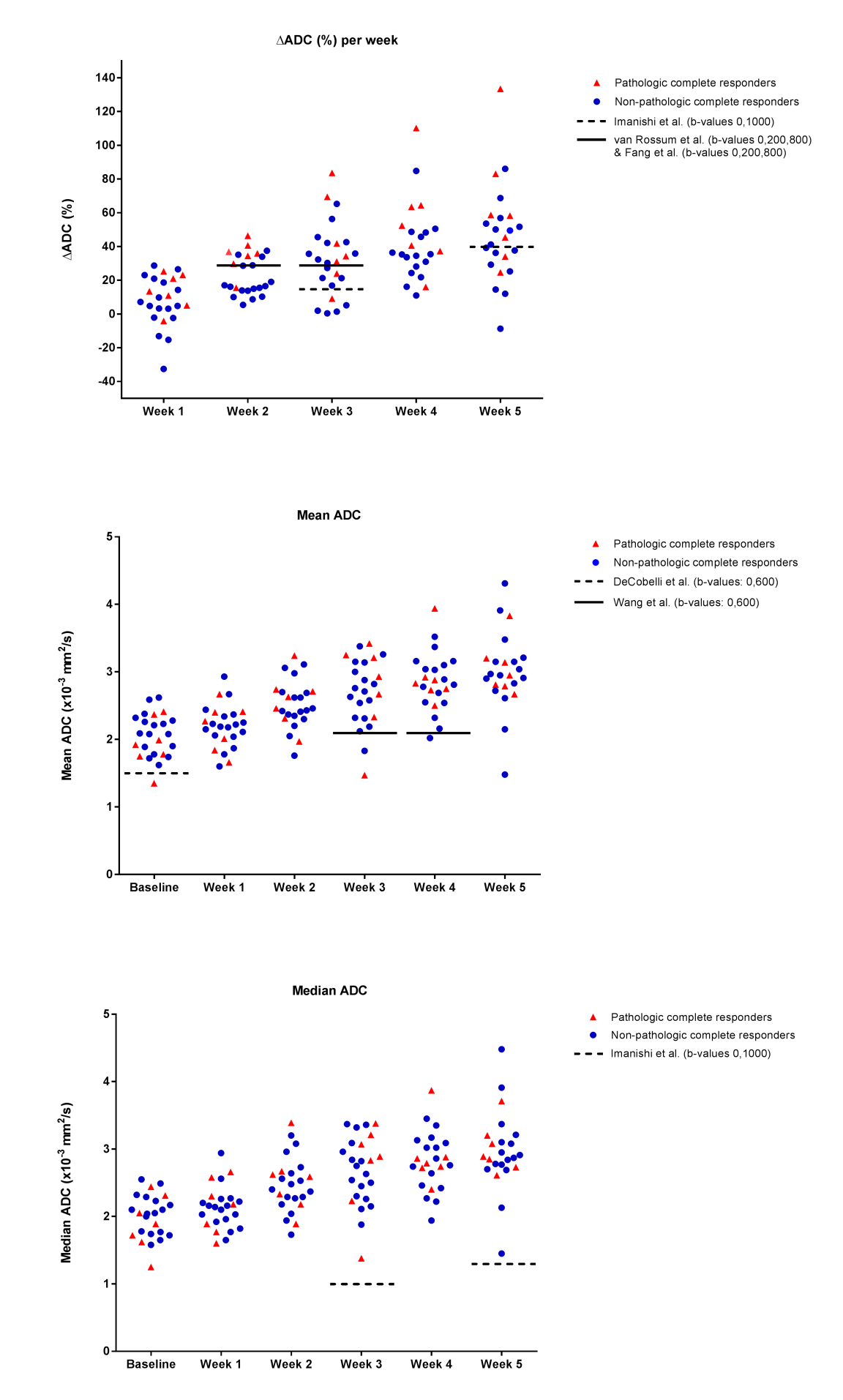

Supplement: Supplementary file 1 — Supplementary methods (radiotherapy, image acquisition details, statistical analyses). Supplementary Table 1. Image acquisition details. Supplementary Table 2. Ridge regression analyses demonstrating the discriminatory value of DW-MRI parameters per week with pathologic complete response (TRG 1) as outcome variable after multiple imputation (20 datasets) of the missing ADC values in week 4 and 5. Supplementary Figure 1. Study design. Supplementary Figure 2. Flowchart. Supplementary Figure 3. Graphic illustration of ΔADC(%) and ADC cut-off points derived from the available literature (represented by the dashed and solid horizontal lines) on DW-MRI in pathologic complete response assessment for esophageal cancer and applied to the current data. (DOCX 277 KB) [file 330_2019_6513_MOESM1_ESM.docx]
